# Von Willebrand factor, ADAMTS13 and mortality in dialysis patients

**DOI:** 10.1186/s12882-021-02420-z

**Published:** 2021-06-16

**Authors:** Gurbey Ocak, Mark Roest, Marianne C. Verhaar, Maarten B. Rookmaaker, Peter J. Blankestijn, Willem Jan W. Bos, Rob Fijnheer, Nathalie C. Péquériaux, Friedo W. Dekker

**Affiliations:** 1 Department of Internal Medicine, Sint Antonius Hospital, Nieuwegein, the Netherlands; 2 Department of Nephrology and Hypertension, University Medical Center Utrecht, Utrecht, the Netherlands; 3 Department of Clinical Epidemiology, Leiden University Medical Center, Leiden, the Netherlands; 4 Synapse Research Institute, Cardiovascular Research Institute, Maastricht, the Netherlands; 5 Department of Internal Medicine, Leiden University Medical Center, Leiden, the Netherlands; 6 Department of Internal Medicine, Meander Medical Center, Amersfoort, the Netherlands; 7 Department of Clinical Chemistry and Hematology, Jeroen Bosch Hospital, ‘s-Hertogenbosch, the Netherlands

**Keywords:** Dialysis, Von Willebrand Factor, ADAMTS13, Mortality, Epidemiology

## Abstract

**Background:**

Von Willebrand Factor (VWF) multimers are cleaved into smaller and less coagulant forms by the metalloprotease ADAMTS13. The aim of this study was to investigate the association between VWF and ADAMTS13 and mortality in dialysis patients.

**Methods:**

We prospectively followed 956 dialysis patients. VWF levels and ADAMTS13 activity were measured. Cox proportional hazard analyses were used to calculate hazard ratios (HRs) with 95 % confidence intervals (CIs) to investigate the association between quartiles of VWF levels and ADAMTS13 activity and all-cause mortality. HRs were adjusted for age, sex, body mass index, cardiovascular disease, dialysis modality, primary kidney disease, use of antithrombotic medication, systolic blood pressure, albumin, C-reactive protein and residual GFR.

**Results:**

Of the 956 dialysis patients, 288 dialysis patients died within three years (mortality rate 151 per 1000 person-years). The highest quartile of VWF as compared with lower levels of VWF was associated with a 1.4-fold (95 %CI 1.1–1.8) increased mortality risk after adjustment. The lowest quartile of ADAMTS13 activity as compared with other quartiles was associated with a 1.3-fold (95 %CI 1.0-1.7) increased mortality risk after adjustment. The combination of the highest VWF quartile and lowest ADAMTS13 quartile was associated with a 2.0-fold (95 %CI 1.3-3.0) increased mortality risk as compared with the combination of the lowest VWF quartile and highest ADAMTS13 quartile.

**Conclusions:**

High VWF levels and low ADAMTS13 activity were associated with increased mortality risks in dialysis patients.

**Supplementary Information:**

The online version contains supplementary material available at 10.1186/s12882-021-02420-z.

## Background

Von Willebrand Factor (VWF) is an important protein for platelet function [1, 2]. Furthermore, VWF carries factor VIII and protects factor VIII from degradation [1, 2]. VWF is mainly produced in endothelial cells and the activity of VWF depends largely on its multimer size. The activity of VWF increases with the multimer size. VWF is cleaved in the circulation by ADAMTS13, which converts large VWF multimers into smaller portions [3, 4]. The combination of a low ADAMTS13 activity with a high concentration of VWF is likely to be prothrombotic with an increased risk of cardiovascular diseases and death. In addition, a severe deficiency of ADAMTS13 could lead to thrombotic thrombocytopenic purpura (TTP), which is a potentially fatal thrombotic disorder [5].

In the general population, increased VWF and decreased ADAMTS13 have been associated with myocardial infarction [6–9] and ischemic stroke, [10–13] probably by inducing a prothrombotic tendency [14]. Two previous studies found that increased VWF levels were associated with increased mortality risks in dialysis patients [15, 16]. In our cohort, we previously showed that the highest quartile of VWF levels were associated with an 1.8-fold increased mortality risk as compared with the lowest quartile of VWF levels [15]. Another cohort study including 55 dialysis patients showed a 2.6-fold increased mortality risk for each one-point increment in VWF levels in IU/ml [16]. However, these studies did not investigate the association between mortality and ADAMTS13 or the combination of ADAMTS13 and VWF. There is only limited information about the combination of increased VWF and decreased ADAMTS13 and the association with all-cause and cause-specific mortality (cardiovascular and non-cardiovascular outcomes). One previous population-based cohort study among individuals aged ≥ 55 years showed that patients with the highest quartile of VWF levels and the lowest quartile of ADAMTS13 levels had a 1.6-fold increased mortality risk as compared with patients with the lowest quartile of VWF levels and the highest quartile of ADAMTS13.[17] In the dialysis population, it is not known whether VWF and ADAMTS13 are associated with all-cause and cause-specific (cardiovascular and non-cardiovascular) mortality.

Dialysis patients have a prothrombotic tendency and have highly increased mortality risks [18]. The investigation of ADAMTS13 and VWF could be of clinical relevance for several reasons. There has been a debate on the effectiveness of vitamin K antagonist in dialysis patients with atrial fibrillation [19, 20]. It could be that patients with low ADAMTS13 levels and high VWF levels reflecting highly prothrombotic patients may benefit from antithrombotic therapy.

The aim of this study was to investigate the association between all-cause and cause-specific (cardiovascular and non-cardiovascular) mortality for VWF and ADAMTS13 in dialysis patients.

## Methods

### Study population

NECOSAD (Netherlands Cooperative Study on the Adequacy of Dialysis) is a multicenter, prospective cohort study, in which 38 dialysis centers throughout the Netherlands participated. Incident chronic hemodialysis and peritoneal dialysis patients were included between 1997 and 2007 if they were ≥ 18 years and had no previous kidney replacement therapy. For this study, we only included patients twelve months after the start of dialysis. Patients were followed till the time of death or censored due to kidney transplantation or loss to follow-up or until March 2010. The study was approved by all local medical ethics committees and all patients gave written informed consent.

### Demographic and clinical data

Data on age, sex, body mass index and primary kidney disease were collected at the start of dialysis treatment. Primary kidney disease was classified according to the codes of the European Renal Association-European Dialysis and Transplant Association (ERA-EDTA) [21]. We grouped patients into seven classes of primary kidney disease: glomerulonephritis, interstitial nephritis, cystic kidney disease, renal vascular disease, diabetes mellitus, multisystem disease and other kidney diseases. Data on comorbidities, use of antithrombotic medication (vitamin K antagonists or antiplatelet drugs), smoking, blood pressure and laboratory data were collected at twelve months after the start of dialysis, which was defined as baseline. C-reactive protein (CRP), albumin, urea and creatinine were routinely measured in the dialysis centers at twelve months after start of dialysis. Residual glomerular filtration rate (GFR) was calculated as the mean of creatinine and urea clearance corrected for body surface area (ml/min per 1.73 m2). Von Willebrand factor levels and ADAMTS13 activity were measured twelve months after the start of dialysis using in house enzyme-linked immunosorbent assays in blood sampled before a dialysis session. Polyclonal rabbit antihuman VWF antibodies were used for catching and tagging (DakoCytomation, Glostrup, Denmark). ADAMTS13 activity was measured in a kinetic assay with the Fluorescence Resonance Energy Transfer Substrate VWF 73.

### Mortality

Causes of death were classified according to the ERA-EDTA codes, which is a standardized classification of death causes in dialysis patients [21]. Death causes were grouped into cardiovascular and non-cardiovascular mortality. Cardiovascular mortality was defined as death due to myocardial ischemia and infarction (code 11), cardiac arrest/sudden death (code 15), cardiac failure/fluid overload/pulmonary edema (codes 14,16,18), hyperkalemia/hypokalemia (code 12,17), pulmonary embolism (code 21), stroke (code 22), hemorrhage from ruptured vascular aneurysm (code 26), mesenteric infarction (code 29) and cause of death uncertain/unknown (code 0). All other deaths were defined as non-cardiovascular mortality.

### Statistical analysis

Continuous variables are presented as median and interquartile range (IQR). Categorical variables are presented as percentages. Patients were categorized based on quartiles of VWF levels and ADAMTS13 activity. Hazard ratios (HRs) with 95 % confidence intervals (95 % CIs) for all-cause and cause-specific mortality within three years of follow-up were calculated using Cox proportional hazard regression analysis. Hazard ratios with 95 % CIs were calculated for the highest quartile of VWF levels as compared with the other quartiles. Furthermore, hazard ratios with 95 % Cis were calculated for the lowest quartile of ADAMTS13 activity as compared with the other quartiles. To investigate the association between the combination of VWF levels and ADAMTS13 activity, we calculated hazard ratios with 95 % CIs for the combination of the highest VWF quartile (p > 75) and lowest ADAMTS13 activity quartile (p ≤ 25) as compared with other combinations of VWF quartiles and ADAMTS13 quartiles. In a sensitivity analysis, we stratified the results for hemodialysis and peritoneal dialysis patients.

Hazard ratios were adjusted for age, sex, body mass index, cardiovascular disease, smoking, dialysis modality, primary kidney disease, use of antithrombotic medication, systolic blood pressure, albumin and CRP levels and residual GFR. To account for missing data of body mass index, albumin and CRP levels and residual GFR, missing values were imputed ten times using the fully conditional specification [22–25]. All analyses have been done in SPSS statistical software version 23.0 (IBM SPSS Statistics).

## Results

### Baseline characteristics

Baseline characteristics of the 956 dialysis patients are shown in Table 1. The median age was 62.9 years, 40.2 % were female, 15.4 % had diabetes mellitus as primary kidney disease and 41.1 % used antithrombotic medication (vitamin K antagonists or antiplatelet drugs). The mean VWF was 20.5 ug/ml and the mean activity of ADAMTS13 was 40.9 %. Supplemental Table 1 shows the baseline characteristics stratified for VWF quartiles and supplemental Table 2 shows the baseline characteristics stratified for ADAMTS13 quartiles. Patients with highest quartile of VWF or lowest quartile of ADAMTS13 had an increased age, cardiovascular disease, diabetes mellitus and an increased CRP as compared with highest quartile of VWF or lowest quartile of ADAMTS13, respectively. During a mean follow-up of 2.0 years, 288 dialysis patients died within three years (mortality rate 151 per 1000 person-years).

**Table 1:** Baseline characteristics

	Total (***N***=956)	
Age in years	62.9	(51.0-72.3)
Female sex	384	(40.2%)
Body mass index^a^ (kg/m^2^)	24.5	(22.3-27.2)
Systolic blood pressure (mmHg)	141	(129-155)
Cardiovascular disease	343	(35.9%)
Smoking	210	(22.0%)
Antithrombotic medication	393	(41.1%)
Antiplatelet agents	234	(24.5%)
Anticoagulant drugs	153	(16.0%)
Both	6	(0.6%)
Dialysis modality		
Hemodialysis	685	(71.7%)
Peritoneal dialysis	271	(28.3%)
Primary Kidney Disease		
Glomerulonephritis	133	(13.9%)
Interstitial nephritis	109	(11.4%)
Cystic kidney disease	114	(11.9%)
Vascular disease	175	(18.3%)
Diabetes mellitus	147	(15.4%)
Multisystem disease	63	(6.6%)
Other	501	(52.4%)
Residual GFR^b^ (ml/min)	1.9	(0.6-3.7)
Albumin^c^ (g/L)	37	(33-40)
C-reactive protein^d^ (mg/L)	6	(3-15)

^a^Missing in 18 patients

^b^Missing in 187 patients

^c^Missing in 29 patients

^d^Missing in 338 patients

### Von Willebrand factor quartiles and mortality

**Fig. 1:**
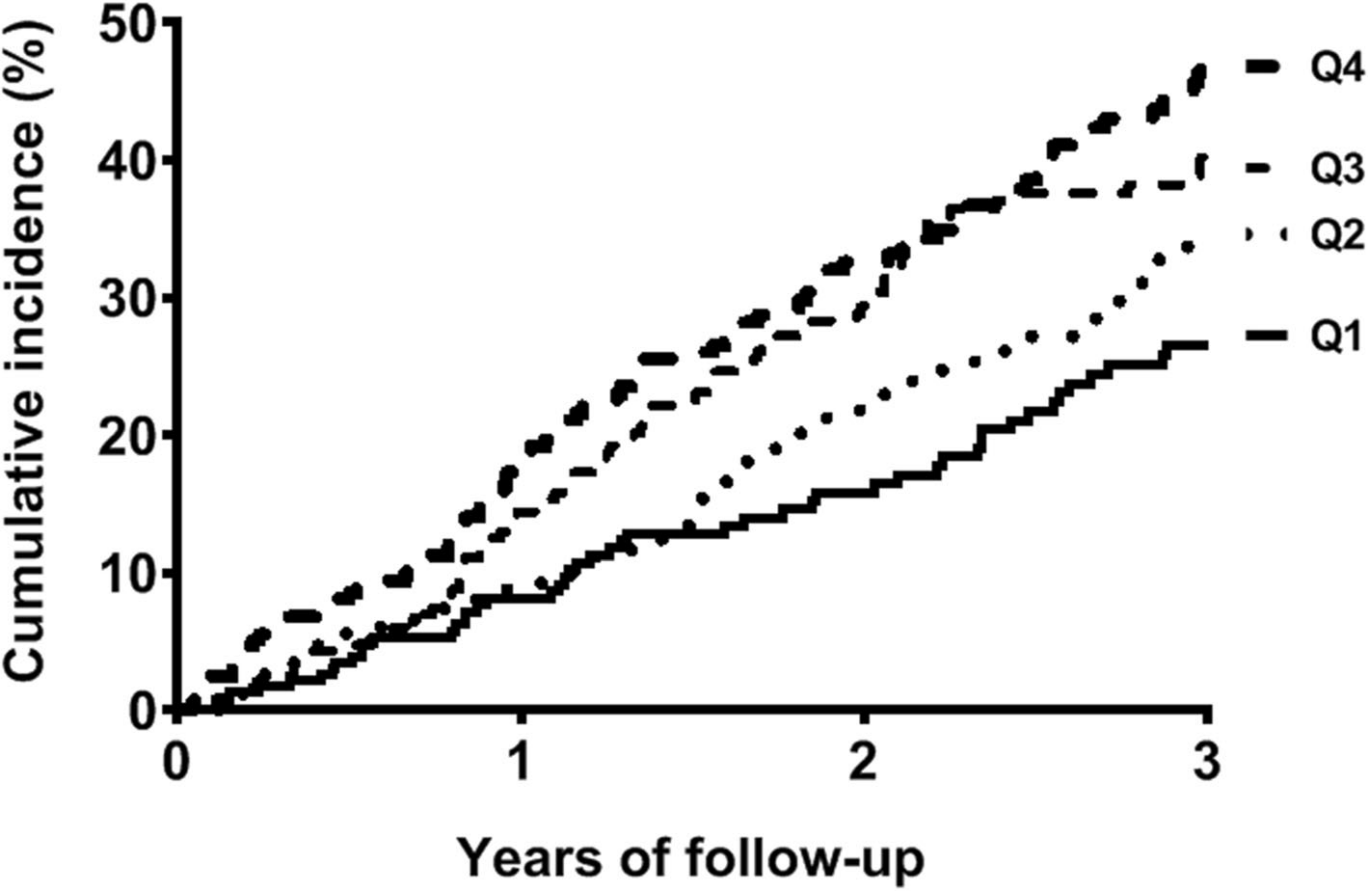
Kaplan–Meier survival curves for Von Willebrand factor quartiles

The mean level of VWF was 36.2 ug/ml in the highest quartile of VWF and 9.4 ug/mL in the lowest quartile. Figure 1 shows the inverted Kaplan–Meier survival curves of VWF quartiles for mortality within three years. The cumulative incidence of mortality increased with increasing VWF levels. The cumulative incidence of mortality after three years was 26.6 % for the lowest VWF quartile and 48.4 % for the highest VWF quartile. The highest quartile of VWF as compared with lower levels of VWF was associated with a 1.4-fold (95 %CI 1.1–1.8) increased mortality risk after adjustment for age, sex, body mass index, cardiovascular disease, smoking, dialysis modality, primary kidney disease, use of antithrombotic medication, systolic blood pressure, albumin levels, CRP levels and residual GFR (Table 2). The highest quartile of VWF as compared with lower levels of VWF was associated with a 1.3-fold (95 % CI 0.9–1.9) increased cardiovascular mortality risk and a 1.5-fold (95 % CI 1.0-2.2) increased non-cardiovascular mortality risk after adjustment.

**Table 2:** Von Willebrand factor quartiles and mortality

	Total ***N***=956	Mean level (ug/mL)	Mortality rate per 1000 patient years	Crude HR (95% CI)		Adjusted^**a**^ HR (95% CI)	
**All-cause mortality**							
Quartile 1	239	9.4	99	*reference*		*reference*	
Quartile 2	239	15.4	129	1.3	(0.9-1.9)	1.2	(0.8-1.7)
Quartile 3	239	21.0	172	1.7	(1.2-2.5)	1.5	(1.1-2.2)
Quartile 4	239	36.2	209	2.1	(1.5-3.0)	1.8	(1.2-2.6)
***Quartile 4 versus Quartile 1-3***				***1.6***	***(1.2-2.0)***	***1.4***	***(1.1-1.8)***
**Cardiovascular mortality**							
Quartile 1	239	9.4	51	*reference*		*reference*	
Quartile 2	239	15.4	73	1.4	(0.8-2.3)	1.3	(0.8-2.1)
Quartile 3	239	21.0	82	1.6	(1.0-2.6)	1.4	(0.9-2.4)
Quartile 4	239	36.2	102	2.0	(1.2-3.3)	1.7	(1.0-2.8)
***Quartile 4 versus Quartile 1-3***				***1.5***	***(1.1-2.1)***	***1.3***	***(0.9-1.9)***
**Non-cardiovascular mortality**							
Quartile 1	239	9.4	47	*reference*		*reference*	
Quartile 2	239	15.4	56	1.2	(0.7-2.1)	1.1	(0.6-1.9)
Quartile 3	239	21.0	90	1.9	(1.2-3.2)	1.6	(1.0-2.8)
Quartile 4	239	36.2	107	2.3	(1.4-3.7)	1.9	(1.1-3.3)
***Quartile 4 versus Quartile 1-3***				***1.7***	***(1.2-2.4)***	***1.5***	***(1.0-2.2)***

^a^Adjusted for age, sex, body mass index, cardiovascular disease, dialysis modality, smoking, primary kidney disease, use of antithrombotic medication, systolic blood pressure, albumin, C-reactive protein and residual GFR

### ADAMTS13 quartiles and mortality

**Fig. 2:**
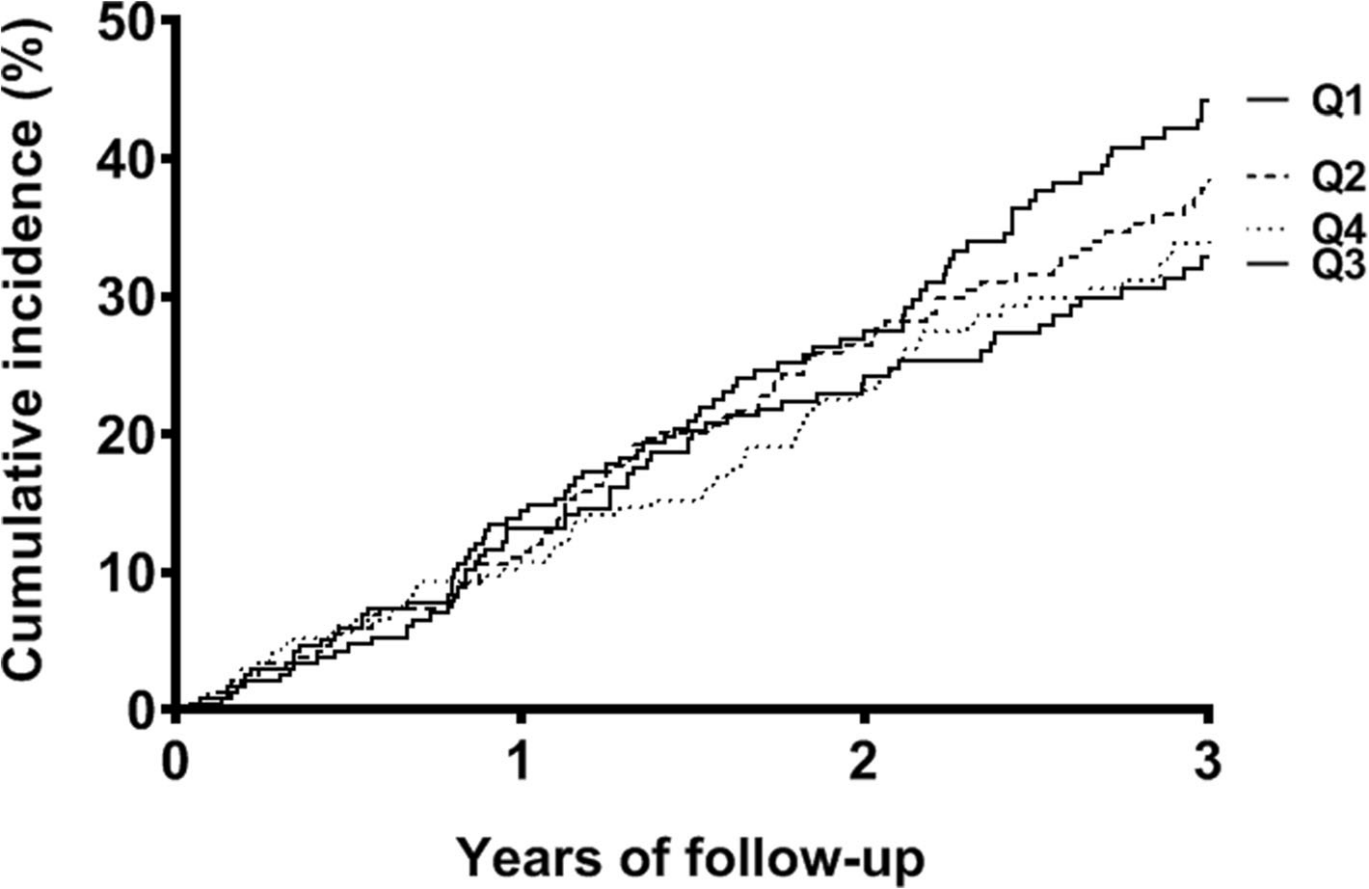
Kaplan–Meier survival curves for ADAMTS13 quartiles

The mean activity of ADAMTS13 was 16.7 % in the lowest quartile of ADAMTS13 and 68.1 % in the highest quartile of ADAMTS13. Figure 2 shows the inverted Kaplan–Meier survival curves of ADAMTS quartiles for mortality within three years. The cumulative incidence of mortality was highest for the lowest ADAMTS13 quartile (44.2 %) and lowest for the highest ADAMTS13 quartile (34.6 %). The lowest quartile of ADAMTS13 as compared with higher quartiles of ADAMTS13 was associated with a 1.3-fold (95 %CI 1.0-1.7) increased mortality risk after adjustment (Table 3). The lowest quartile of ADAMTS13 as compared with higher levels of ADAMTS13 was associated with a 1.2-fold (95 % CI 0.9–1.8) increased cardiovascular mortality risk and a 1.4-fold (95 % CI 1.0–2.0) increased non-cardiovascular mortality risk after adjustment.

**Table 3:** ADAMTS13 quartiles and mortality

	Total ***N***=956	Mean level (activity %)	Mortality rate per 1000 patient years	Crude HR (95% CI)		Adjusted^**a**^ HR (95% CI)	
**All-cause mortality**							
Quartile 1	239	16.7	181	1.3	(1.0-1.8)	1.2	(0.9-1.7)
Quartile 2	239	33.0	156	1.1	(0.8-1.6)	0.9	(0.7-1.3)
Quartile 3	239	45.7	132	1.0	(0.7-1.4)	0.9	(0.6-1.2)
Quartile 4	239	68.1	136	*reference*		*reference*	
***Quartile 1 versus Quartile 2-4***				***1.3***	***(1.1-1.7)***	***1.3***	***(1.0-1.7)***
**Cardiovascular mortality**							
Quartile 1	239	16.7	88	1.2	(0.8-1.8)	1.1	(0.7-1.8)
Quartile 2	239	33.0	80	1.1	(0.7-1.7)	0.9	(0.6-1.4)
Quartile 3	239	45.7	63	0.8	(0.5-1.4)	0.8	(0.5-1.3)
Quartile 4	239	68.1	75	*reference*		*reference*	
***Quartile 1 versus Quartile 2-4***				***1.2***	***(0.9-1.6)***	***1.2***	***(0.9-1.8)***
**Non-cardiovascular mortality**							
Quartile 1	239	16.7	92	1.5	(1.0-2.4)	1.3	(0.8-2.2)
Quartile 2	239	33.0	76	1.3	(0.8-2.0)	1.0	(0.6-1.6)
Quartile 3	239	45.7	69	1.1	(0.7-1.9)	1.0	(0.6-1.6)
Quartile 4	239	68.1	61	*reference*		*reference*	
***Quartile 1 versus Quartile 2-4***				***1.4***	***(1.0-1.9)***	***1.4***	***(1.0-2.0)***

^a^Adjusted for age, sex, body mass index, cardiovascular disease, smoking, dialysis modality, primary kidney disease, use of antithrombotic medication, systolic blood pressure, albumin, C-reactive protein and residual GFR

### Combination of ADAMTS13 and von Willebrand factor and mortality

In patients with both high VWF levels (highest quartile, p ≥ 75) and low ADAMTS13 activity (lowest quartile, p ≤ 25), the risk of all-cause mortality (HR 2.0; 95 % CI 1.3-3.0) was increased compared with low VWF levels (lowest quartile, p < 75) and high ADAMTS13 activity (highest quartile, p > 25) after adjustment (Table 4). High VWF levels and low ADAMTS13 activity as compared with low VWF levels and high ADAMTS13 activity were associated with a 1.8-fold (95 % CI 1.0-3.2) increased cardiovascular mortality risk and a 2.1-fold (95 % CI 1.2–3.7) increased non-cardiovascular mortality risk.

**Table 4:** Combination of ADAMTS13 and von Willebrand factor and mortality

	Total ***N***=956	Mortality rate per 1000 patient years	Crude HR (95% CI)		Adjusted^**a**^ HR (95% CI)	
**All-cause mortality**						
VWF p<75 AND ADAMTS13 p>25	549	125	*reference*		*reference*	
VWF p<75 AND ADAMTS13 p≤25	168	159	1.3	(0.9-1.7)	1.2	(0.9-1.7)
VWF p≥75 AND ADAMTS13 p>25	168	197	1.6	(1.2-2.1)	1.3	(1.0-1.8)
VWF p≥75 AND ADAMTS13 p≤25	71	241	2.0	(1.3-2.9)	2.0	(1.3-3.0)
**Cardiovascular mortality**						
VWF p<75 AND ADAMTS13 p>25	549	65	*reference*		*reference*	
VWF p<75 AND ADAMTS13 p≤25	168	79	1.2	(0.8-1.9)	1.2	(0.7-1.8)
VWF p≥75 AND ADAMTS13 p>25	168	99	1.5	(1.0-2.3)	1.2	(0.8-1.9)
VWF p≥75 AND ADAMTS13 p≤25	71	112	1.8	(1.0-3.1)	1.8	(1.0-3.2)
**Non-cardiovascular mortality**						
VWF p<75 AND ADAMTS13 p>25	549	60	*reference*		*reference*	
VWF p<75 AND ADAMTS13 p≤25	168	79	1.3	(0.8-2.1)	1.3	(0.8-2.1)
VWF p≥75 AND ADAMTS13 p>25	168	99	1.7	(1.1-2.5)	1.5	(0.9-2.3)
VWF p≥75 AND ADAMTS13 p≤25	71	128	2.2	(1.3-3.7)	2.1	(1.2-3.7)

^a^Adjusted for age, sex, body mass index, cardiovascular disease, smoking, dialysis modality, primary kidney disease, use of antithrombotic medication, systolic blood pressure, albumin, C-reactive protein and residual GFR

### Sensitivity analysis

As a sensitivity analysis, we calculated HRs stratified for hemodialysis and peritoneal dialysis. In hemodialysis patients, the highest quartile of VWF as compared with lower levels of VWF was associated with a 1.5-fold (95 %CI 1.1-2.0) increased mortality risk and the lowest quartile of ADAMTS13 as compared with higher quartiles of ADAMTS13 was associated with a 1.3-fold (95 %CI 1.0-1.8) increased mortality risk after adjustment. High VWF levels and low ADAMTS13 activity as compared with low VWF levels and high ADAMTS13 activity were associated with a 2.2-fold (95 % CI 1.4–3.5) increased mortality risk in hemodialysis patients. In peritoneal dialysis patients, risk estimates for the highest quartile of VWF (HR 1.3, 95 % CI 0.7–2.3), lowest quartile of ADAMTS13 (HR 1.4, 95 % CI 0.7–2.5) and the combination of high VWF levels and low ADAMTS13 activity (HR 1.7, 95 % CI 0.7–4.3) were increased, but did not reach statistical significance.

## Discussion

In this prospective cohort study of 956 incident dialysis patients, increased VWF levels and decreased ADAMTS13 activity were associated with an increased risk of mortality. We found a 1.4-fold increased all-cause mortality risk for the highest quartile of VWF as compared with lower levels of VWF and a 1.3-fold increased mortality risk for the lowest quartile of ADAMTS13 activity as compared with other quartiles. The mortality rates were increased for both cardiovascular and non-cardiovascular causes. We found the highest mortality rates for patients who had both high VWF levels and low ADAMTS13 activity. The combination of the highest VWF quartile and lowest ADAMTS13 quartile was associated with a 2.0-fold increased mortality risk as compared with the combination of the lowest VWF quartile and highest ADAMTS13 quartile.

In the general population, most previous studies focused on the association between VWF, ADAMTS13 and non-fatal cardiovascular outcomes (myocardial infarction [6–9] or stroke [10–13]). In a meta-analysis that investigated the association between VWF, ADAMTS13 and cardiovascular outcomes, [14] most of the studies found an increased risk of myocardial infarction or stroke. Risk estimates for increased VWF levels ranged from a 0.9-fold to a 4.7-fold increased risk of myocardial infarction and ranged from a 1.0-fold to a 6.7-fold increased risk of stroke. Risk estimates for decreased ADAMTS13 activity ranged from a 0.5-fold to an 8.2-fold increased risk of myocardial infarction and ranged from a 1.7-fold to a 7.3-fold increased risk of stroke [14]. The ranges in the risk estimates were wide, probably because of differences in study design and study population of these studies. We also found increased risk estimates of cardiovascular mortality for high VWF levels as compared with low VWF levels (hazard ratio of 1.7) and low ADAMTS13 activity as compared with high ADAMTS13 activity (hazard ratio of 1.2), which is within the range of the previous studies.

Studies investigating the association between all-cause mortality and increased ADAMTS13 activity and decreased VWF levels are limited. One previous study investigated the association between mortality and decreased ADAMTS13 activity and increased VWF levels in the general population [17]. As in our study, the previous study showed increased all-cause mortality risks for high VWF levels and low ADAMTS13 activity. The highest quartile of VWF as compared with the lowest quartile of VWF was associated with a 1.2-fold increased all-cause mortality risk and the lowest quartile of ADAMTS13 as compared with higher quartiles of ADAMTS13 was associated with a 1.5-fold increased mortality risk after adjustment. Furthermore, it was shown that high VWF levels (highest quartile, p ≥ 75) and low ADAMTS13 activity (lowest quartile, p ≤ 25) were associated with a 1.6-fold increased all-cause mortality risk as compared with low VWF levels (lowest quartile, p < 75) and high ADAMTS13 activity (highest quartile, p > 25) after adjustment, which is in line the 2.0-fold increased risk found in our study. We and others found that increased VWF levels were associated with increased mortality risks in dialysis patients [15, 16]. However, these studies did not investigate the association between mortality and ADAMTS13 or the combination of ADAMTS13 and VWF.

An explanation for the associations between mortality and high VWF levels and low ADAMTS13 activity could be a prothrombotic tendency. In a normal situation, VWF multimers are cleaved in the circulation by the metalloprotease ADAMTS13, which converts large VWF multimers into smaller and less procoagulant forms [3, 4]. However, in case of high VWF levels and low ADAMTS13 activity, thrombus formation and thrombus growth could be exaggerated by the prothrombotic tendency. This could eventually lead to an increased cardiovascular mortality risk. Another important note is that in our dialysis population, mean levels of VWF were higher (20.5 ug/ml versus 10 ug/ml) and mean activity levels of ADAMTS13 were lower (40.9 % versus 100 %) than in the general population [17]. Also other studies found higher VWF levels and lower ADAMTS13 activity in dialysis patients than non-dialysis patients [26, 27]. Therefore, a prothrombotic tendency could be highly prevalent in dialysis patients. Increased VWF levels and lower ADAMTS13 activity in dialysis patients as compared with the general population could be a reflection of a high prevalence of comorbidities, including cardiovascular diseases and diabetes mellitus. We did not investigate genetic determinants for VWF levels and ADAMTS13 activity. In our study, besides increased risk estimates for cardiovascular mortality, risk estimates were also increased for non-cardiovascular mortality. It could be that VWF and ADAMTS13 are also involved in progression of other diseases than arterial thrombosis (myocardial infarction and stroke) leading to an increased non-cardiovascular mortality. There are some studies showing a role of VWF and ADAMTS13 in pulmonary diseases, [28] sepsis [29, 30] and cancer [31, 32]. The exact mechanisms behind these findings are not known.

The main strength of this study was the large and well-defined prospective cohort of dialysis patients with available data on many patient characteristics and laboratory measurements, which allowed us to investigate the association between VWF, ADAMTS13 and mortality. Another strong point of the study is that all measurements of VWF and ADAMTS13 have been done twelve months after initiation of dialysis. This reduces the risk that measurements were affected by fluctuations commonly observed at the commencement of dialysis in the acute phase. Our study also has several potential limitations. An important limitation in the association between VWF, ADAMTS13 and mortality is the possibility of residual confounding. In our analyses, we took this into account by correcting for multiple confounders, but this cannot exclude possible residual confounding. Therefore, it could be that high VWF and low ADAMTS13 are markers for other underlying diseases that we did not adjust for. Another limitation of our study was the limited power in the subgroup analysis with peritoneal dialysis patients. However, risk estimates were more or less similar with hemodialysis patients. Furthermore, we had no information about the type, dose or indication of anticoagulants and antiplatelet agents to investigate the role of these factors in the association between mortality VWF and ADAMTS13. Finally, we could not investigate the association between VWF and ADAMTS13 and mortality in a subgroup of patients with atrial fibrillation, since we had no information about the presence of atrial fibrillation in our cohort. Dialysis patients have highly increased risks of atrial fibrillation [20] and VWF and ADAMTS13 have been associated with worse outcomes in patients with atrial fibrillation [33, 34]. Therefore, atrial fibrillation could play an important role in the association between VWF and ADAMTS13 and mortality in dialysis patients.

Decreased ADAMTS13 and increased VWF levels are relevant in TTP, which clinically manifests itself as thrombocytopenia, micro-angiopathic hemolytic anemia, fever, neurological symptoms and renal insufficiency due to the formation of microthrombi [5]. Our finding that the combination of increased VWF and decreased ADAMTS13 is associated with an increased mortality risk could also have clinical implications in dialysis patients. There has been a debate on the effectiveness of antithrombotic therapy in dialysis patients, since studies did not show a decreased risk of ischemic stroke in dialysis patients who used vitamin K antagonists as compared with patients without vitamin K antagonist use [19, 20]. Furthermore, ischemic stroke models have weak predictive performances in incident dialysis patients [35]. Patients with low ADAMTS13 levels and high VWF levels reflecting highly prothrombotic patients may be a subgroup of patients who could benefit from antithrombotic therapy.

## Conclusions

In summary, we have shown that increased VWF levels and decreased ADAMTS13 activity were associated with an increased risk of mortality in dialysis patients.

## Supplementary Information

**Additional file 1:** **Supplemental Table 1**: Baseline characteristics stratified for Von Willebrand factor quartiles

**Additional file 2:** **Supplemental Table 2**: Baseline characteristics stratified for ADAMTS13 quartiles

## Data Availability

The datasets used and analyzed during the current study are available from the corresponding author on reasonable request.

## Abbreviations

VWF: Von Willebrand Factor
TTP: Thrombotic thrombocytopenic purpura
GFR: Glomerular filtration rate
CRP: C-reactive protein
ERA-EDTA: European Renal Association-European Dialysis and Transplant Association
