# Supplementary material for: Von Willebrand factor, ADAMTS13 and mortality in dialysis patients
Source: BMC Nephrol. 2021 Jun 16;22:222. doi: 10.1186/s12882-021-02420-z (PMC8207579; doi:10.1186/s12882-021-02420-z)
Supplement: Supplementary file 1 — Additional file 1: Supplemental Table 1: Baseline characteristics stratified for Von Willebrand factor quartiles [file 12882_2021_2420_MOESM1_ESM.docx]

**Supplemental Table 1. Baseline characteristics stratified for Von Willebrand factor quartiles**

|  | | **Quartile 1^a^**  **(N=239)** | | **Quartile 2^b^**  **(N=239)** | | **Quartile 3^c^**  **(N=239)** | | **Quartile 4^d^**  **(N=239)** | |
| --- | --- | --- | --- | --- | --- | --- | --- | --- | --- |
| Age in years | | 58.4 | (48.2-70.3) | 62.5 | (50.0-71.7) | 64.7 | (52.0-74.0) | 65.0 | (54.3-73.8) |
| Female sex | | 87 | (36.4%) | 95 | (39.7%) | 106 | (44.4%) | 96 | (40.2%) |
| Body mass index (kg/m^2^) | | 24.1 | (21.8-26.6) | 24.7 | (22.5-27.3) | 24.5 | (22.3-26.8) | 24.6 | (22.6-28.0) |
| Systolic blood pressure (mmHg) | | 146 | (132-158) | 140 | (130-153) | 143 | (130-157) | 136 | (120-150) |
| Cardiovascular disease | | 82 | (34.3%) | 85 | (35.6%) | 86 | (36.0%) | 90 | (37.7%) |
| Smoking | | 55 | (23.0%) | 55 | (23.0%) | 50 | (20.9%) | 50 | (20.9%) |
| Antithrombotic medication | | 87 | (36.4%) | 99 | (41.4%) | 135 | (56.5%) | 136 | (56.9%) |
| Dialysis modality | |  |  |  |  |  |  |  |  |
|  | Hemodialysis | 203 | (84.9%) | 189 | (79.1%) | 169 | (70.7%) | 124 | (51.9%) |
|  | Peritoneal dialysis | 36 | (15.1%) | 50 | (20.9%) | 70 | (29.3%) | 115 | (48.1%) |
| Primary Kidney Disease | |  |  |  |  |  |  |  |  |
|  | Glomerulonephritis | 46 | (19.2%) | 21 | (8.8%) | 29 | (12.1%) | 37 | (15.5%) |
|  | Interstitial nephritis | 28 | (11.7%) | 38 | (15.9%) | 29 | (12.1%) | 14 | (5.9%) |
|  | Cystic kidney disease | 33 | (13.8%) | 32 | (13.4%) | 25 | (10.5%) | 24 | (10.0%) |
|  | Vascular disease | 33 | (13.8%) | 51 | (21.3%) | 48 | (20.1%) | 43 | (18.0%) |
|  | Diabetes mellitus | 32 | (13.4%) | 38 | (15.9%) | 35 | (14.6%) | 42 | (17.6%) |
|  | Multisystem disease | 9 | (3.8%) | 18 | (7.5%) | 20 | (8.4%) | 16 | (6.7%) |
|  | Other | 58 | (24.3%) | 41 | (17.2%) | 53 | (22.2%) | 63 | (26.4%) |
| Residual GFR (ml/min) | | 2.2 | (1.0-3.8) | 2.1 | (0.8-4.2) | 1.7 | (0.2-3.6) | 1.8 | (0.1-3.6) |
| Albumin (g/L) | | 37 | (34-40) | 37 | (33-40) | 37 | (33-39) | 36 | (32-39) |
| C-reactive protein (mg/L) | | 5 | (3-10) | 6 | (3-15) | 8 | (4-16) | 8 | (3-17) |

^a^Missings: body mass index in 3 patients, residual GFR in 45 patients, albumin in 7 patients, C-reactive protein in 83 patients

^b^Missings: body mass index in 2 patients, residual GFR in 49 patients, albumin in 10 patients, C-reactive protein in 72 patients

^c^Missings: body mass index in 3 patients, residual GFR in 43 patients, albumin in 6 patients, C-reactive protein in 83 patients

^d^Missings: body mass index in 10 patients, residual GFR in 50 patients, albumin in 6 patients, C-reactive protein in 100 patients
