# Supplementary material for: Von Willebrand factor, ADAMTS13 and mortality in dialysis patients
Source: BMC Nephrol. 2021 Jun 16;22:222. doi: 10.1186/s12882-021-02420-z (PMC8207579; doi:10.1186/s12882-021-02420-z)
Supplement: Supplementary file 2 — Additional file 2: Supplemental Table 2: Baseline characteristics stratified for ADAMTS13 quartiles [file 12882_2021_2420_MOESM2_ESM.docx]

**Supplemental Table 2. Baseline characteristics stratified for ADAMTS13 quartiles**

|  | | **Quartile 1^a^**  **(N=239)** | | **Quartile 2^b^**  **(N=239)** | | **Quartile 3^c^**  **(N=239)** | | **Quartile 4^d^**  **(N=239)** | |
| --- | --- | --- | --- | --- | --- | --- | --- | --- | --- |
| Age in years | | 63.1 | (52.1-72.3) | 65.3 | (53.4-73.5) | 62.2 | (51.4-71.2) | 60.5 | (48.1-71.6) |
| Female sex | | 93 | (38.9%) | 83 | (34.7%) | 140 | (58.6%) | 130 | (54.4%) |
| Body mass index (kg/m^2^) | | 24.3 | (22.2-27.2) | 24.5 | (22.2-27.3) | 24.5 | (22.1-27.2) | 24.6 | (22.7-27.2) |
| Systolic blood pressure (mmHg) | | 140 | (127-154) | 142 | (128-155) | 143 | (130-158) | 140 | (130-154) |
| Cardiovascular disease | | 91 | (38.1%) | 104 | (43.5%) | 85 | (35.6%) | 63 | (26.4%) |
| Smoking | | 50 | (20.9%) | 52 | (21.8%) | 58 | (24.3%) | 50 | (20.9%) |
| Antithrombotic medication | | 90 | (37.7%) | 108 | (45.2%) | 106 | (44.4%) | 89 | (37.2%) |
| Dialysis modality | |  |  |  |  |  |  |  |  |
|  | Hemodialysis | 164 | (68.6%) | 189 | (79.1%) | 182 | (76.2%) | 150 | (62.8%) |
|  | Peritoneal dialysis | 75 | (31.4%) | 50 | (20.9%) | 57 | (23.8%) | 89 | (37.2%) |
| Primary Kidney Disease | |  |  |  |  |  |  |  |  |
|  | Glomerulonephritis | 29 | (12.1%) | 29 | (12.1%) | 41 | (17.2%) | 34 | (14.2%) |
|  | Interstitial nephritis | 21 | (8.8%) | 22 | (9.2%) | 36 | (15.1%) | 30 | (12.6%) |
|  | Cystic kidney disease | 31 | (13.0%) | 21 | (8.8%) | 28 | (11.7%) | 34 | (14.2%) |
|  | Vascular disease | 44 | (18.4%) | 55 | (23.0%) | 33 | (13.8%) | 43 | (18.0%) |
|  | Diabetes mellitus | 40 | (16.7%) | 33 | (13.8%) | 33 | (13.8%) | 41 | (17.2%) |
|  | Multisystem disease | 17 | (7.1%) | 19 | (7.9%) | 13 | (5.4%) | 14 | (5.9%) |
|  | Other | 57 | (23.8%) | 60 | (25.1%) | 55 | (23.0%) | 43 | (18.0%) |
| Residual GFR (ml/min) | | 1.8 | (0.5-3.8) | 1.7 | (0.3-3.2) | 2.1 | (0.7-4.4) | 2.2 | (0.9-3.9) |
| Albumin (g/L) | | 37 | (33-40) | 37 | (33-40) | 36 | (33-39) | 37 | (34-40) |
| C-reactive protein (mg/L) | | 8 | (3-15) | 7 | (3-16) | 6 | (3-14) | 6 | (3-13) |

^a^Missings: body mass index in 5 patients, residual GFR in 54 patients, albumin in 6 patients, C-reactive protein in 83 patients

^b^Missings: body mass index in 8 patients, residual GFR in 62 patients, albumin in 10 patients, C-reactive protein in 90 patients

^c^Missings: body mass index in 1 patient, residual GFR in 35 patients, albumin in 4 patients, C-reactive protein in 77 patients

^d^Missings: body mass index in 4 patients, residual GFR in 36 patients, albumin in 9 patients, C-reactive protein in 88 patients
